# Starved Nerves and Famished Teeth

**Published:** 1889-10

**Authors:** 


### STARVED NERVES AND FAMISHED TEETH.

It may not be generally known that the same element required to nourish the bones, are also equally necessary for the maintenance of the nervous tissues of the body, the brain, and the nerves. Bad nerves and bad teeth are neither an infrequent nor an accidental combination of ailments. The same conditions of body which lead to lowered nerve tone, lead to decay of the teeth, whether the cause be a disturbance of digestion which prevents the proper assimilation of the " salts " (the bone and nerve building elements of body), or a deficient supply of these important elements in the dietary. Premature decay of the teeth is an ominous outlook for an individual ; it means premature decay of brain and nerves as well ; it means an early loss of the energy and bouyancy of youth. In view of these facts, there is a sad future before the American people. The condition of the teeth of the average American is such that it has been asserted that a hundred years hence, at the rate at which deposits of gold in human teeth are now taking place, there will be found more gold in the cemeteries of the United States than in the mines of Colorado. However this may be, certain it is that the young man or woman of twenty who has thirty-two, or even twenty-eight, sound teeeth is an exceptional individual. Plenty of boys and girls of sixteen or seventeen years are wearing artificial teeth.

It is worth while to inquire into the cause of this premature decay. There are, doubtless, two important causes, overlooking several minor ones. These are, first, the introduction of superfine preparations of the grains in modern times ; and second, the general physical decline of the race. That portion of the grain which until within a few years the farmer fed to his hogs contains in largest proportion the elements needed for the nourishment of brains and bones. It is no wonder, then, that the farmer raised fine hogs and puny children. The accumulated effects of this starvation of the body, as regards the class of elements needed for teeth and nerves, for several generations back, is now seen in the premature decay of these structures Dentists and lunatic asylums flourish and multiply beyond all precedent. The peripatetic dentist is no longer seen. He finds work enough at home. The victims of crumbling grinders are not widely scattered through communities, but constitute the majority.

The remedy for this state of things, so far as food is concerned, is to be found in the use of whole-grain preparations. Oatmeal, unbolted wheat flour, known in this country and Germany as graham flour, whole-wheat flour, rye and corn bread, and the legumes, peas and beans, afford salts in abundance. But these foods must be digested and assimilated as well as eaten. The American disease, dyspepsia, is doubtless largely dependent on the general lowered nerve tone of the American people, which is a natural result of a century of high-pressure living, and is a serious obstacle in the way of the improvement of our famished teeth and nerves. Salts cannot be assimilated until they have first been digested. A first step toward improved digestion will be in the abandonment of tea, ices, pastry, greasy foods, and the adoption of simpler habits in diet. Then we must have more out-of-door exercise, more muscle work, and less excitement of brains and nerve. We do not say less brain work, but less excitement, less worry, less indulgence in such nerve-exhausting recreations as balls, theaters, horse-racing, and progressive-euchre parties.

We are often told that "the world moves." Assuredly it does. It moves too fast. It rushes, it whirls, it gyrates like a western cyclone. We should be gratefnl if some one would tell us, and support the assertion by facts, that "the world pauses;" at any rate, that its headlong destructive speed is slowing down a little. Our teeth are crumbling to atoms under the pressure of our bad habits, dietetic and otherwise; our nerves are snapping with the tension of our stimulated life; our brains are reeling with the intoxication of excitement. It is time for us to pause, and give attention to the requirements of nature's simple laws, before we become a soft-brained and toothless race.—*Good Health.*

